# Functional Analysis of the Promoter Regions of Two Apoptosis-Related Genes (*Bcl-2* and *Cycs*) and Their Regulation by Zn in Yellow Catfish

**DOI:** 10.3390/ijms22126291

**Published:** 2021-06-11

**Authors:** Yang He, Tao Zhao, Fang Chen, Changchun Song, Chongchao Zhong, Zhi Luo

**Affiliations:** Laboratory of Molecular Nutrition for Aquatic Economic Animals, Fishery College, Huazhong Agricultural University, Wuhan 430070, China; heyang1997@webmail.hzau.edu.cn (Y.H.); zhaotao2017@webmail.hzau.edu.cn (T.Z.); chenfang95@webmail.hzau.edu.cn (F.C.); songchangchun@webmail.hzau.edu.cn (C.S.); zhongchongchao@webmail.hzau.edu.cn (C.Z.)

**Keywords:** apoptosis, promoter analysis, transcriptional regulation, Zn, vertebrates

## Abstract

B-cell lymphoma 2 (Bcl-2) and cytochrome c (Cycs) are two important proteins relevant to cellular apoptosis. In this study, we characterized the functions of the promoter regions of two apoptosis-related genes, *Bcl-2* and *Cycs*, in yellow catfish *Pelteobagrus fulvidraco*. We obtained a 1989 bp *Bcl-2* promoter and an 1830 bp *Cycs* promoter and predicted several key transcription factor binding sites (TFBSs) on the promoters, such as Kruppel-like factor 4 (KLF4), signal transducer and activator of transcription factor 3 (STAT3), forkhead box O (FOXO), metal-responsive element (MRE) and hepatocyte nuclear factor 1α (HNF-1α). Zinc (Zn) increased the activities of the *Bcl-2* promoter but decreased the activities of the *Cycs* promoter. Metal-responsive transcription factor 1 (MTF-1) and HNF-1α directly bound with *Bcl-2* and *Cycs* promoters, and they positively regulated the activity of the *Bcl-2* promoter but negatively regulated the activity of the *Cycs* promoter. Zn promoted the binding ability of HNF-1α to the *Bcl-2* promoter but decreased its binding ability to the *Cycs* promoter. However, Zn had no significant effect on the binding capability of MTF-1 to the regions of *Bcl-2* and *Cycs* promoters. Zn upregulated the mRNA and total protein expression of Bcl-2 but downregulated the mRNA and total protein expression of Cycs. At the same time, Annexin V–FITC/PI staining showed that Zn significantly reduced the apoptosis of primary hepatocytes. For the first time, our study provides evidence for the MRE and HNF-1α response elements on the *Bcl-2* and *Cycs* promoters, offering new insight into the mechanism by which Zn affects apoptosis in vertebrates.

## 1. Introduction

As a naturally occurring and evolutionarily conserved physiological process, apoptosis plays an important role in tissue development, physiology and homeostasis [1]. Cytochrome c is an important component of the apoptosome, which initiates the caspase cascade to induce apoptosis [2]. In contrast, Bcl-2 is an antiapoptotic protein and protects cells from apoptosis by inhibiting the cytochrome c release from the mitochondria [3,4]. Therefore, Bcl-2 and Cycs play very essential roles in the regulation of apoptosis. However, *Bcl-2* and *Cycs* promoters have been studied extensively in mammals but have rarely been reported in fish [5,6]. Zn is an important micronutrient for vertebrates, including fish [7,8,9]. However, excessive Zn may cause toxic effects for organisms [10]. At present, the study of Zn toxicity mainly focuses on growth depression, oxidative stress and histopathological damage. Meanwhile, Zn is an important antiapoptotic factor [11,12]. Previous studies show that Zn inhibits apoptosis through three pathways: inhibiting the activity of Ca^2+^/Mg^2+^-dependent endonuclease responsible for nuclear DNA fragmentation, inhibiting the activity of effector caspase-3 and increasing the Bcl-2/Bax ratio [13,14,15]. Studies also show that Zn treatment upregulates Bcl-2 expression and downregulates Cycs expression [16,17]. Recently, we found that Zn exposure influenced the transcription levels of *Bcl-2* and *Cycs* in *Pelteobagrus fulvidraco* [8,18]. However, the molecular mechanism for the transcriptional regulation of *Bcl-2* and *Cycs* in response to Zn remains to be explored.

MTF-1 is an important Zn finger transcription factor that regulates cell adaptation to Zn by binding with the MRE of its target gene promoter [19]. Research also suggests that MTF-1 mediates the regulation of apoptosis [20]. HNF1, predominantly expressed in the liver, plays an important role in regulating the gene expression of liver tissues [21]. HNF1 has also been reported to have antiapoptotic effects [22,23,24]. These studies indicate that MTF-1 and HNF-1α are associated with apoptosis. However, the direct link between these transcriptional factors and apoptosis remained to be further explored.

As a kind of freshwater fish, yellow catfish *Pelteobagrus fulvidraco* is of high market value due to its delicious meat quality and is widely cultured in China and other Asian countries [25]. In our laboratory, the cDNA full-length sequences of apoptosis-related genes *Bcl-2* and *Cycs* have been cloned in yellow catfish [18,26]. However, so far, the transcriptional regulatory mechanism of Zn on these two genes remains unknown. Our experiment was a continuation of the study on the relationship between apoptosis and Zn in yellow catfish. In this study, the regions of *Bcl-2* and *Cycs* promoters were identified, and the binding sites of MTF-1 and HNF-1α in their promoter regions were deciphered in yellow catfish. Furthermore, the transcriptional regulation of *Bcl-2* and *Cycs* by Zn was determined. In addition, the apoptosis of hepatocytes in yellow catfish treated with Zn was detected by flow cytometry. Our study offers a novel insight into the molecular mechanism by which Zn regulates apoptosis in vertebrates.

## 2. Results

### 2.1. The Identification of Promoters

**Figure 1:**
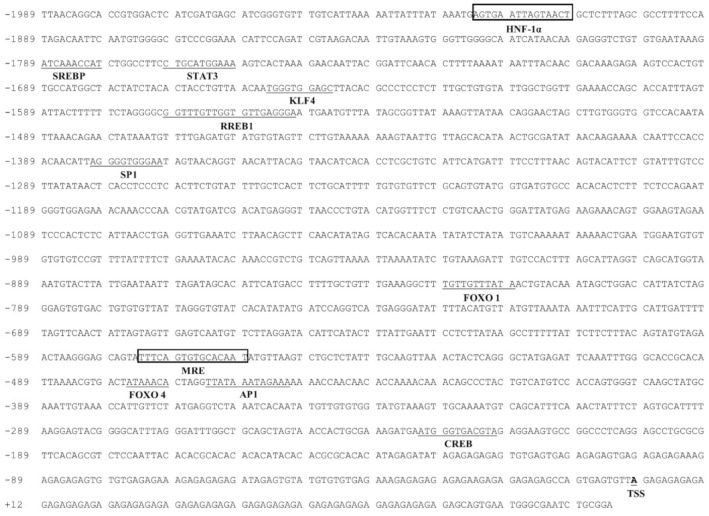
Nucleotide sequence of yellow catfish *Bcl-2* promoter. Numbers are relative to the transcription start site (TSS) (+1). The putative TFBSs are underlined. The highlighted sequences show putative TFBSs of MRE and HNF-1α.

**Figure 2:**
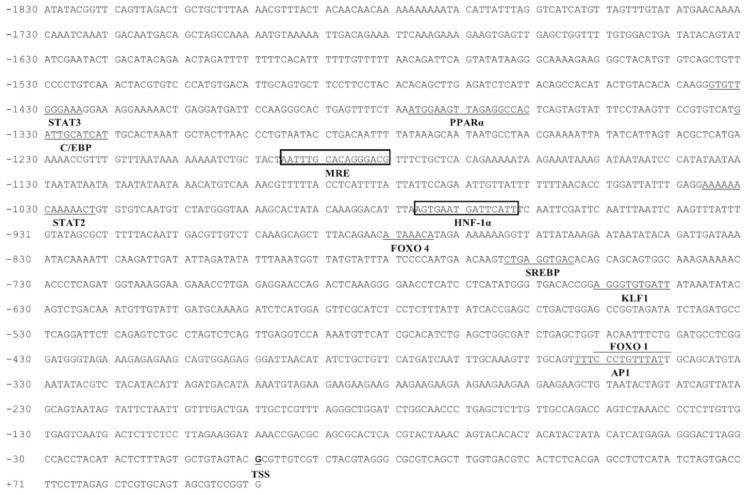
Nucleotide sequence of yellow catfish *Cycs* promoter. Numbers are relative to the TSS (+1). The putative TFBSs are underlined. The highlighted sequences show putative TFBSs of MRE and HNF-1α.

We cloned the 1989 bp *Bcl-2* promoter (Figure 1) and 1830 bp *Cycs* promoter (Figure 2). The transcription starting sites (TSSs) of *Bcl-2* and *Cycs* were identified. The first nucleotide of each TSS was designated as +1. On the *Bcl-2* promoter, some TFBSs were predicted, such as cAMP response element-binding protein (CREB) (−221/−232 bp), sterol regulatory element-binding proteins (SREBP) (−1780/−1789 bp), KLF4 (−1646/−1655 bp), ras-responsive element-binding protein 1 (RREB1) (−1551/−1570 bp), FOXO1 (−819/−829 bp) and FOXO4 (−470/−476 bp), specificity protein 1 (SP1) (−1371/−1381 bp) and STAT3 (−1760/−1770 bp). The putative binding sites of MRE (−559/−574 bp) and HNF−1α (−1910/−1924 bp) were also found in the *Bcl-2* promoter.

On the *Cycs* promoter, several important TFBSs, such as FOXO1 (−341/−351 bp), KLF1 (−641/−651 bp), SREBP (−755/−764 bp), FOXO4 (−875/−881 bp), STAT2 (−1023/−1036 bp), CCAAT/enhancer-binding protein (C/EBP) (−1321/−1331 bp), peroxisome proliferator-activated receptor alpha (PPARα) (−1361/−1378 bp) and STAT3 (−1425/−1435 bp) were discovered on its promoter region. Meantime, MRE (−1181 bp/−1196 bp) and HNF−1α (−963/−977 bp) binding sites were also found in the *Cycs* promoter.

### 2.2. The 5′-Sequence Deletion Analysis

**Figure 3:**
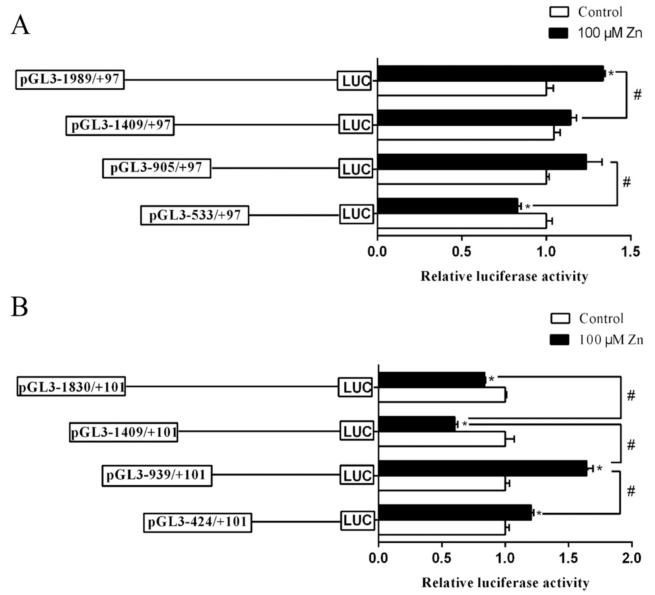
The 5′ unidirectional deletion assays for *Bcl-2* and *Cycs* promoter regions after Zn incubation. (**A**) *Bcl-2* promoter; (**B**) *Cycs* promoter. Values are means ± SEM (*n* = 3 independent biological experiments). *p* value was calculated by Student’s *t* tests. Asterisk (*) indicates significant differences in relative luciferase activities between Zn-treated group and the control; hash symbol (^#^) indicates significant differences in relative luciferase activities between two promoter regions (*p* < 0.05).

To investigate the response of the two promoters to Zn, HEK293T cells were incubated with 100 μM ZnSO_4_, followed by 5’-sequence deletion analysis. For *Bcl-2* promoter, compared to the control, Zn treatment decreased the luciferase activity within the deletion plasmids of −533/+97 bp but increased the activity within −1989/+97 bp, indicating that Zn had a regulatory effect on *Bcl-2* promoter. Moreover, for different fragments of plasmids treated with Zn, the luciferase activity of the −905/+97 bp region was significantly higher than that of −533/+97 bp, and the luciferase activity of −1989/+97 bp was also significantly higher than that of −1409/+97 bp, indicating that the two regions from −533 bp to −905 bp and from −1409 bp to −1989 bp have positive regulatory elements (Figure 3A). 

For the *Cycs* promoter, compared to the control, Zn incubation significantly increased the luciferase activities of −424/+101 bp and −939/+101 bp fragments but decreased the luciferase activity of −1409/+101 bp and −1830/+101 bp fragments. For different sizes of promoter regions, Zn incubation also increased the luciferase activities of the regions from −424 bp to −939 bp and from −1409 bp to −1830 bp but reduced the relative activity of the region from −939 bp to −1409 bp, indicating potential positive regulatory elements in the regions of −424 bp to −939 bp and −1409 bp to −1830 bp, and negative elements between −939 bp and −1409 bp in response to Zn, respectively (Figure 3B).

### 2.3. Site-Mutation Analysis 

**Figure 4:**
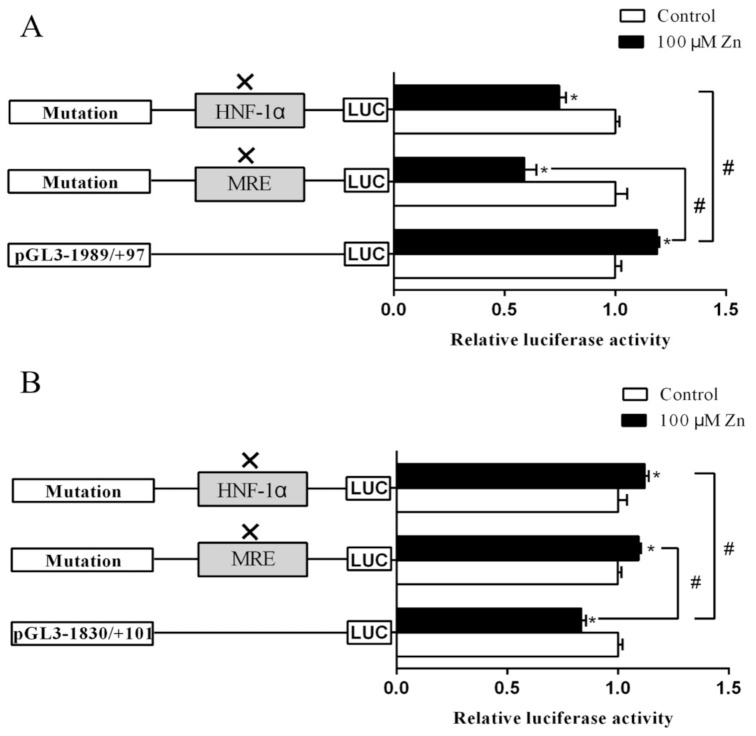
Assays of predicted binding sites after site-directed mutagenesis. (**A**) The luciferase activity assays of predicted MRE and HNF-1α sites after site-directed mutagenesis in the *Bcl-2* promoter. (**B**) The luciferase activity assays of predicted MRE and HNF-1α sites after site-directed mutagenesis in the *Cycs* promoter. Values are means ± SEM (*n* = 3 independent biological experiments). *p* value was calculated by Student’s *t* tests. Asterisk (*) indicates significant differences in relative luciferase activities between Zn-treated group and the control; hash symbol (^#^) indicates significant differences in relative luciferase activities between two promoters (*p* < 0.05).

For the *Bcl-2* promoter, Zn increased the luciferase activity at the site pGl3−1989/+97 bp. However, the mutation of the −559/−574 bp MRE site (Mut-Bcl-2-MRE) and −1910/−1924 bp HNF−1α site (Mut-Bcl-2-HNF-1α) alleviated the increase of luciferase activity induced by Zn, indicating these two sites are involved in the transcriptional response of *Bcl-2* gene to Zn. Zn reduced the luciferase activity of −559/−574 bp site mutant plasmid (Mut-Bcl-2-MRE) compared to the control, indicating that the MRE site plays a positive regulatory role in *Bcl-2* transcription. Similarly, −1910/−1924 bp site mutant plasmid (Mut-Bcl-2-HNF-1α) also alleviated the Zn-induced increase in luciferase activity. Thus, these results reflect that −559/−574 bp MRE and −1910/−1924 bp HNF-1α sites play positive regulatory roles in Zn-induced *Bcl-2* transcription (Figure 4A).

For the *Cycs* promoter, compared to wild-type (WT) pGl3 −1830/+101 bp plasmid, the mutation of −1181/−1196 bp MRE site (Mut-Cycs-MRE) and −963/−977 bp HNF-1α site (Mut-Cycs-HNF-1α) significantly increased the luciferase activities after Zn incubation, indicating that these two sites are important for Zn-induced *Cycs* transcription. Compared with the WT pGl3−1830/+101 bp vector, the mutation of −1181/−1196 bp MRE site (Mut-Cycs-MRE) and −963/−977 bp HNF-1α site (Mut-Cycs-HNF-1α) increased the luciferase activity in the Zn-treated group, suggesting that these two sites negatively regulate Zn-induced *Cycs* transcription (Figure 4B).

### 2.4. Analysis of the Functional Binding Sites Based on Electrophoretic Mobility Shift Assay (EMSA)

**Figure 5:**
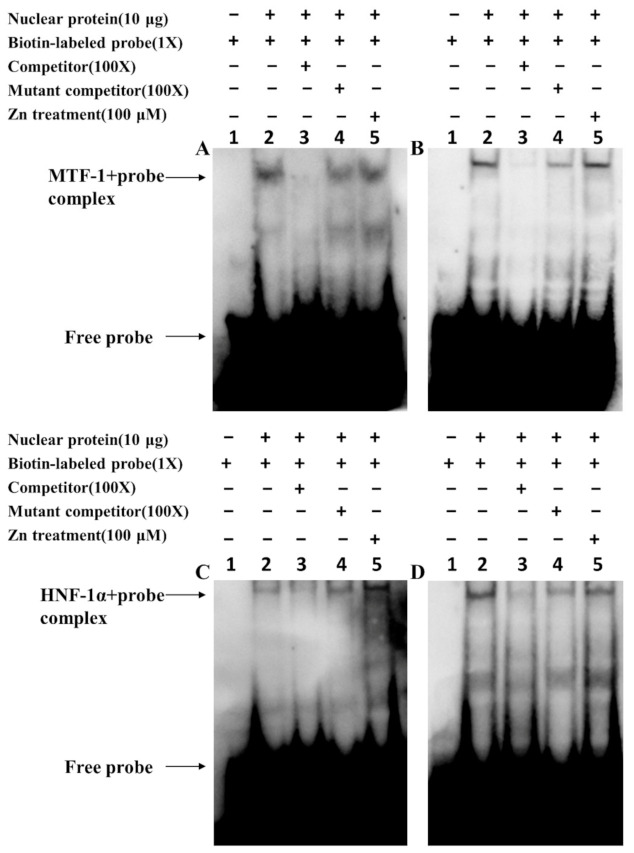
Electrophoretic mobility shift assay (EMSA) analysis of predicted TFBSs on the yellow catfish *Bcl-2* and *Cycs* promoters: (**A**) −559/−574 bp MRE site of *Bcl-2* promoter; (**B**) −1181/−1196 bp MRE site of *Cycs* promoter; (**C**) −1910/−1924 bp HNF-1α of *Bcl-2* promoter; (**D**) −963/−977 bp HNF-1α site of *Cycs* promoter. The numbers 1–5 represent the five different lanes.

According to the above results of our site-mutation analysis, the −559/−574 bp for MRE binding and −1910/−1924 bp for HNF-1α binding on the *Bcl-2* promoter and the −1181/−1196 bp for MRE and −963/−977 bp for HNF-1α binding on *Cycs* promoter were considered to be functional. EMSA analysis was used to further confirm these potential binding sites. For the *Bcl-2* promoter, we used the MRE binding sequence as the probe and found that the 100-fold unlabeled binding site of MRE (located at −559 to −574 bp) competed for the binding, but the 100-fold unlabeled Mut-Bcl-2-MRE sequence did not compete for the protein binding, suggesting that MTF-1 could bind with this region (Figure 5A). For the *Cycs* promoter, the −1181/−1196 bp MRE site competed for the binding with the nuclear protein; however, the 100-fold unlabeled Mut-Cycs-MRE site did not compete for the nuclear protein with the labeled probe, indicating that −1181/−1196 bp MRE sequence of the *Cycs* promoter could bind with the nuclear protein (Figure 5B). Compared to the control, there was no significant change in the band brightness after Zn treatment, indicating that the MRE sites of *Bcl-2* and *Cycs* promoters did not interact with Zn significantly (Figure 5A,B).

Meanwhile, for the *Bcl-2* promoter, when the HNF-1α binding sequence was used as the probe, we found that the 100-fold unlabeled HNF-1α binding site (located at −1910 to −1924 bp) competed for the binding; however, the 100-fold unlabeled Mut-Bcl-2-HNF-1α sequence did not compete with the labeled probe for the nuclear protein (Figure 5C). Further, Zn increased the brightness of the band, suggesting the Zn mediates the regulation of *Bcl-2* by the −1910/−1924 bp HNF-1α sites at the transcriptional level. For the *Cycs* promoter, the −963/−977 bp HNF-1α binding sequence was bound by HNF-1α (Figure 5D). Meanwhile, Zn decreased the brightness of bands, suggesting that Zn mediates the transcriptional regulation of *Cycs* by the −963/−977 bp HNF-1α site. These results indicate that the MRE and HNF-1 α binding sites of *Bcl-2* and *Cycs* promoters are functional binding sites. 

### 2.5. mRNA and Protein Expression Induced by Zn

**Figure 6:**
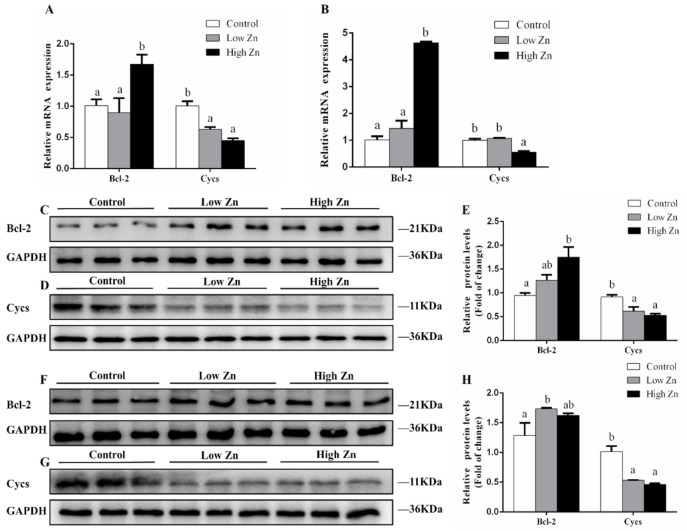
Effects of Zn incubation on Bcl-2 and Cycs expression in the hepatocytes of yellow catfish: (**A**) *Bcl-2* and *Cycs* gene expression normalized to *β-actin* and *rpl7* at 24 h; (**B**) *Bcl-2* and *Cycs* gene expression normalized to *β-actin* and *ubce* at 48 h; (**C**) Bcl-2 total protein expression at 24 h; (**D**) Cycs total protein expression at 24 h; (**E**) the relative densities of total Bcl-2 and Cycs protein at 24 h were measured by Image-Pro Plus; (**F**) Bcl-2 total protein expression at 48 h; (**G**) Cycs total protein expression at 48 h; (**H**) the relative density of total Bcl-2 and Cycs protein at 48 h were measured by Image-Pro Plus. Values are means ± SEM (*n* = 3), and experiments were repeated three times. *p* value was calculated by one-way ANOVA and further post hoc Duncan’s multiple range testing. Values without the same letter indicate significant difference among three treatments (*p* < 0.05).

To demonstrate the effect of Zn on Bcl-2 and Cycs, we measured the gene and protein expression under Zn treatment at 24 and 48 h. At 24 h, among three groups, the *Bcl-2* mRNA levels were highest for the high Zn group, but there was no significant difference between the control and the low Zn group, and *Cycs* mRNA expression was highest for the control (Figure 6A). At 48 h, the level of *Bcl-2* mRNA increased in high Zn treatment, indicating that Zn upregulates *Bcl-2* expression in a concentration-dependent manner. Among the three groups, the *Cycs* mRNA level was the lowest for the high Zn group (Figure 6B).

For the protein expression, at 24 h, Bcl-2 protein level increased with the Zn concentration; the protein level of Cycs tended to decline with Zn concentration (Figure 6C–E). At 48 h, Zn significantly upregulated the Bcl-2 protein level. Both low and high Zn levels significantly decreased the protein expression of Cycs (Figure 6F–H). 

Overall, these results prove that Zn upregulates the expression of Bcl-2 mRNA and protein and downregulates the expression of Cycs, indicating that they play antiapoptotic roles.

### 2.6. Analysis of the Effect of Zn on Apoptosis 

**Figure 7:**
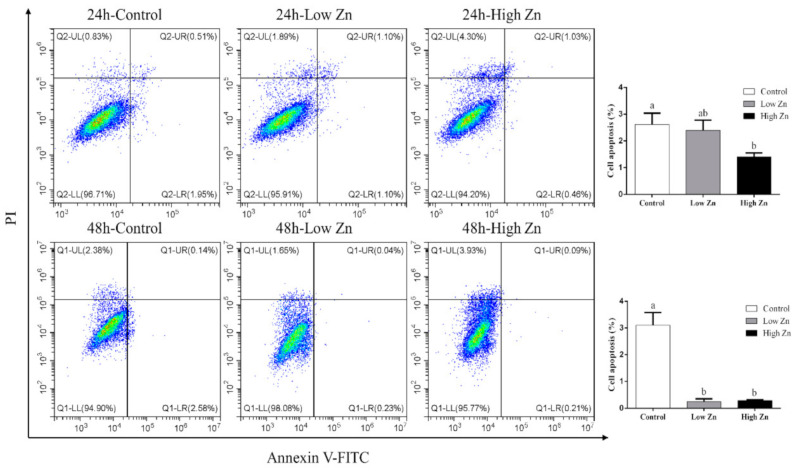
Effects of Zn incubation on apoptosis in the hepatocytes of yellow catfish. Only one representative of three independent experiments is presented for each group. The number of apoptotic cells was calculated as the sum of Q-LR and Q-UR (Q-UL, PI-positive and annexin-negative; Q-LL, both annexin- and PI-negative; Q-LR, annexin-positive and PI-negative; Q-UR, both annexin- and PI-positive). Values are means ± SEM (*n* = 3). *p* value was calculated by one-way ANOVA and further post hoc Duncan’s multiple range testing. Values without the same letter indicate significant difference among three treatments (*p* < 0.05).

To investigate the effect of Zn on apoptosis, Annexin V–FITC/PI double staining was used to detect apoptotic cells (Figure 7). Early apoptosis was shown by Annexin V–FITC staining (Q-LR), and Annexin V–FITC and PI colabeled cells showed late apoptosis (Q-UR). The final rate of apoptotic cells included early and late apoptotic cells (Q-LR + Q-UR). Compared with the control, high Zn significantly decreased the apoptosis of hepatocytes at 24 h. Similarly, after 48 h incubation, the apoptosis rates in the low Zn and high Zn groups were 0.27% and 0.30%, respectively, significantly lower than that in the control, indicating that Zn could inhibit apoptosis.

## 3. Discussion

Zn mediates the regulation of cell apoptosis [27,28]. Studies show that Zn at different concentrations can inhibit chemically induced apoptosis, and it is also an effective inhibitor of the apoptotic protease caspase-3 [29,30,31,32,33,34]. However, the molecular mechanism by which Zn regulates apoptosis remains to be explored. For the first time, our study found that the promoter regions of apoptosis-related genes *Bcl-2* and *Cycs* have the functional MRE and HNF-1α binding sites that can respond directly to Zn, which provides an important basis for elucidating the transcriptional regulation of apoptosis by Zn.

In the present study, we predicted one activator protein 1 (AP1) and one SP1 in the *Bcl-2* promoter and one AP1 in the *Cycs* promoter, similar to other reports [35,36]. In addition, a series of TFBSs, such as HNF-4α, KLF4, STAT3 and SREBP on the *Bcl-2* promoter and C/EBP, PPARα and FOXO4 in the C*ycs* promoter, were predicted. Studies show that HNF-4α, KLF4, STAT3 and FOXO4 mediate the regulation of cell apoptosis [37,38,39,40]. In addition, SREBP, C/EBP and PPARα are involved in lipid metabolism [41,42,43]. This implies that the transcriptional regulation of *Bcl-2* and *Cycs* is very complex. The identification of TFBSs helps to reveal the mechanism of gene regulation. In the present study, we found that the −559/−574 bp MRE site and −1910/−1924 bp HNF-1α site are crucial for the variations of the *Bcl-2* promoter induced by Zn and play a positive role in regulating the *Bcl-2* promoter in response to Zn. We also found that the −1181/−1196 bp MRE site and −963/−977 bp HNF-1α site are functional binding sites of the *Cycs* promoter and negatively regulate *Cycs* gene in response to Zn. MTF-1 is an intracellular Zn sensor and plays an important role in regulating metal homeostasis by binding to MREs of its target gene promoters, thus activating their expression [19]. Studies show that there are MRE binding sites on the promoters of two Zn transporters (ZIP3 and ZIP8) in yellow catfish, and Zn treatment upregulates the transcriptional activity of the promoters [44]. HNF1, as a liver-enriched transcription factor, plays a key role in regulating the expression of liver-specific genes and has key effects on apoptosis [21,24]. However, to our knowledge, the present study is the first report on the presence of MRE and HNF-1α sites on *Bcl-2* and *Cycs* promoters. Our results indicate that Zn upregulates the activity of the *Bcl-2* promoter and downregulates the activity of the *Cycs* promoter, thereby inhibiting the occurrence of apoptosis. Moreover, we found that apoptosis-related genes have HNF-1α binding sites on their promoters, suggesting its direct link with apoptosis.

In our study, Zn promoted the expression of Bcl-2 protein and gene but inhibited the expression of Cycs. Bcl-2 is an important antiapoptotic factor, and the cytochrome c release is an important marker of cell apoptosis. Cycs could induce apoptosis when it accumulates in the cytoplasm in response to various stress inducers [45,46,47]. Therefore, the increase in Bcl-2 expression and the decrease in Cycs expression indicate that Zn might inhibit the occurrence of apoptosis. Subsequently, we detected the extent of apoptosis with Annexin V–FITC/PI double staining, which provided direct evidence for Zn inhibition of apoptosis. Similarly, Sun et al. [48] pointed out that Zn is a cytoprotective mineral that could inhibit apoptosis. Lin et al. [16] reported that Zn downregulates the levels of cleaved caspase-3 and Bax but increases the level of Bcl-2 in mice. Li et al. [18] showed that Zn exposure upregulates *Bcl-2* expression in yellow catfish. Other studies indicate that Zn inhibits cytochrome c release and Cycs expression [17,27]. Therefore, Zn is an important regulatory factor of cell apoptosis and can inhibit cell apoptosis.

In conclusion, we identified the promotor regions of apoptosis-related genes *Bcl-2* and *Cycs* in yellow catfish and identified the MRE and HNF-1α binding sites in these promoters. Zn mediated the regulation of the transcriptional activity of *Bcl-2* and *Cycs* through MTF-1 and HNF-1α. Zn treatment upregulated *Bcl-2* mRNA and total protein levels but downregulated Cycs expression and significantly inhibited the cell apoptosis of yellow catfish. Our study offers direct evidence for elucidating the mechanism by which Zn mediates apoptosis via the MRE and HNF-1α binding sites on the *Bcl-2* and *Cycs* promoters. For the first time, our study clarifies the underlying mechanism by which Zn regulates the transcriptional activities of *Bcl-2* and *Cycs*, thus revealing a new mechanism of Zn-induced apoptosis.

## 4. Materials and Methods

### 4.1. Ethical Statement

The experiments with yellow catfish followed the Institutional Ethical Guidelines of Huazhong Agricultural University (HZAU) and were approved by the Ethics Committee of HZAU (identification code: Fish-2019-12-21).

### 4.2. Experimental Animals and Reagents

For promoter cloning and functional analysis, yellow catfish were purchased from a local commercial farm (Wuhan, China), and HEK293T cells were obtained from the Cell Resource Center in Fishery College of Huazhong Agricultural University. Lipofectamine 2000 and LightShift Chemiluminescent EMSA Kit were purchased from Invitrogen (Carlsbad, CA, USA). Passive Lysis Buffer and Dual-Luciferase were obtained from Promega (Minneapolis, MN, USA). Dulbecco’s Modified Eagle’s Medium (DMEM), 0.25% trypsin-EDTA and fetal bovine serum (FBS) were obtained from Gibco/Invitrogen (Waltham, MA, USA). Nuclear proteins for EMSA were determined using Pierce BCA protein assay kit (Thermo Fisher Scientific Waltham, MA, USA). Total RNA was isolated with Trizol reagent (Thermo Fisher Scientific Waltham, MA, USA) and transcribed into the cDNA with a Reverse Transcription Kit (Thermo Fisher Scientific Waltham, MA, USA).

### 4.3. Exp. 1: Cloning and Functional Analysis of Bcl-2 and Cycs Promoters

#### 4.3.1. Promoter Cloning, Plasmid Construction

We identified the TSSs of *Bcl-2* and *Cycs* promoters of yellow catfish according to our publications [49,50]. Then, we cloned the sequences of *Bcl-2* and *Cycs* promoters based on the open-access genome of yellow catfish [51] and on those in Xu et al. [49]. Using SacI and Hind III restriction sites, we subcloned the different plasmids containing the sequences of *Bcl-2* and *Cycs* promoters into the pGl3-Basic vectors (Promega, Fitchburg, WI, USA). Using the purified PCR product and pGl3-Basic vectors (Promega, Fitchburg, WI, USA), we generated the luciferase reporter constructs, and we ligated the products by using the ClonExpress II One Step Cloning Kit (Vazyme, Piscataway, NJ, USA). Based on the distance from the TSS, we defined these plasmids as pGl3−1989/+97 of *Bcl-2* and pGl3−1830/+101 of *Cycs*. Using templates of pGl3−1989/+97 of *Bcl-2* vector, we generated the plasmids pGl3−533/+97, pGl3−905/+97, pGl3−1409/+97 and pGl3−1989/+97 of *Bcl-2* containing the unidirectional deletions of the promoters with the Erase-a-Base system (Promega, USA). Similarly, the plasmids pGl3−424/+101, pGl3−969/+101 and pGl3−1409/+101 were produced by using pGl3−1830/+101 of *Cycs* vector as the template. Table S1 lists all primers used for the plasmid construction.

#### 4.3.2. Sequence Analysis

Using MatInspector on 10 September 2020 (http://www.genomatix.de/) and the JASPAR database on 15 November 2020 (http://jaspar.genereg.net/), we predicted the putative transcriptional factor binding sites (TFBSs) of *Bcl-2* and *Cycs* promoters online, and we used the Clustal-W multiple alignment algorithm for the sequence alignment.

#### 4.3.3. Analysis of the 5′-Sequence Deletion of the Bcl-2 and Cycs Promoters

We transfected the plasmid into HEK293T cells and detected the luciferase activities according to the method of Xu et al. [49]. The procedures were as follows: HEK293T cell lines were cultured in the DMEM medium with 10% (*v*/*v*) heat-inactivated FBS (Gibco, Carlsbad, CA, USA) in a SANYO incubator at 37 °C with 5% CO_2_. Lipofectamine 2000 was used as the transfection reagent. The reporter plasmids were cotransfected with the 20 ng pRL TK as the control. After 4 h transfection, the medium was replaced by the DMEM with 10% FBS. Based on our recent studies [19], two Zn concentrations, namely the control (without extra Zn addition) and Zn-treated group (100 μM Zn) were used in this experiment. The concentrations of Zn were selected. Zn was added in the form of ZnSO_4_. After the incubation for 24 h, the cells were lysed and collected. We analyzed the relative luciferase activities by the Dual-Luciferase Reporter Assay System (Promega, Fitchburg, WI, USA).

#### 4.3.4. Site-Mutation Analysis of MRE and HNF-1α Binding Sites on *Bcl-2* and Cycs Promoters

To identify the MRE and HNF-1α binding sites on yellow catfish *Bcl-2* and *Cycs* promoters, we conducted the site-mutation analysis via the QuickChange II Site-Directed Mutagenesis Kit (Vazyme, Piscataway, NJ, USA) according to the manufacturer’s instructions. The site-mutation primers are listed in Table S2. Their mutant plasmids were named Mut-Bcl-2-MRE, Mut-Bcl-2-HNF-1α, Mut-Cycs-MRE and Mut-Cycs-HNF-1α. These constructs and pRL-TK were then cotransfected into HEK293T cells via the Lipofectamine 2000 (Invitrogen, Carlsbad, CA, USA). After 4 h transfection, the medium was replaced by the DMEM with 10% FBS. Two Zn concentrations, namely the control (without extra Zn addition) and the Zn-added group (100 μM Zn), were used in this experiment. After 24 h incubation, the cells were lysed and harvested for the luciferase activities according to the procedure above.

#### 4.3.5. Analysis of the Functional Binding Sites of MTF-1 and HNF-1α on the Bcl-2 and Cycs Promoters Based on EMSA

Analysis of the functional binding sites of MRE and HNF-1α on the Bcl-2 and Cycs promoters followed the method of Xu et al. [49]. After the incubation with or without 100 μM Zn for 24 h, nucleus proteins for EMSA were extracted from HEK293T cells, and their protein concentrations were measured by the BCA method [52]. Oligonucleotide duplex of MRE and HNF-1α was incubated with 10 μg nuclear extracts for 20 min at room temperature, using the Lightshift Chemiluminescent EMSA kit (Invitrogen, Carlsbad, CA, USA). Then, the biotin-labeled probes were added to the system and reacted for 10 min at room temperature. Subsequently, the loading buffer was added and the reaction was conducted for another 10 min before electrophoresis on 6.5% native polyacrylamide gels. The electrophoresis was followed by 10 min of purple coupling, followed by 18 min of sealing solution containing HRP (1:2000), 15 min of sealing solution without HRP, and 4 times of 5 min of washing solution. Finally, Vilber Fusion FX6 Spectra imaging system (Vilber Lourmat, Collegien, France) was used to visualize the binding bands. In this study, 100-fold excess of unlabelled oligonucleotide duplex, in combination with or without the MRE and HNF-1α mutation, was used to perform the competitive analysis. All the oligonucleotide sequences for EMSA are shown in Table S3.

### 4.4. Exp. 2: Effects of Zn Incubation on the Transcriptional Response of Bcl-2 and Cycs

#### 4.4.1. Primary Hepatocyte Culture and Treatments

The primary hepatocytes were isolated from yellow catfish and cultured as previously described [53]. Based on our recent study [19], we designed three Zn concentrations, namely the control, low Zn (50 μM) and high Zn (100 μM). Incubation time was selected based on studies by Liu et al. [54] and Bentayeb et al. [55]. After 24 and 48 h incubation, the cells were collected for real-time quantitative PCR (qPCR), Western blot and flow cytometry analysis.

#### 4.4.2. qPCR

The protocols for the qPCR assay were in agreement with our recent publication [53]. Table S4 lists the primers for each gene. We tested six housekeeping genes, namely *β-actin*, hypoxanthine-guanine phosphoribosyltransferase (*hprt*), beta-2-microglobulin (*b2m*), ribosomal protein L7 (*rpl7*), ubiquitin-conjugating enzyme (*ubce*) and translation elongation factor (*elfa*), to determine their transcriptional stability. We used geNorm software to analyze the most stable two genes as the endogenous regulation, normalized their geometric mean and then calculated the relative expression of genes by 2^−^^△△ct^ method (Center for Medical Genetics, Ghent, Belgium).

#### 4.4.3. Western Blot Analysis

The protein expression was determined via Western blot according to our recent methods [43,52]. The specific primary antibodies anti-Bcl-2 (1:1000, ER1802-97; Huabio, Hangzhou, China), anti-Cycs (1:1000, AB-2757022; Abclonal, Wuhan, China) and anti-GAPDH (1:10,000, #2118; Cell Signaling Technology, Danvers, MA, USA) were used in our study. The Vilber Fusion FX6 Spectra imaging system (Vilber Lourmat, Collegien, France) was used to visualize the protein bands, followed by the quantitation by Image-Pro Plus 6.0.

#### 4.4.4. Flow Cytometry for the Determination of Apoptosis of Hepatocytes

Apoptosis was determined using the Annexin V–FITC/PI double staining method according to the method of Hsu and Yen [56]. In brief, the primary hepatocytes were washed thrice with PBS and centrifuged at 1000 rpm for 5 min. The supernatant was discarded, and 500 μL binding buffer was added for resuspension; then, 5 μL Annexin V–FITC and PI were added and mixed. Next, cells were incubated at room temperature in darkness for 10 min. Finally, cell apoptosis was analyzed by flow cytometry. Ten thousand events were collected for each stained sample.

### 4.5. Statistical Analysis

SPSS 19.0 software was used to perform the statistical analysis. The final results are shown as means ± standard error of mean (SEM). Before the present statistical analysis, we evaluated all data for normality via the Shapiro–Wilk test and for homogeneity of variance via Bartlett’s test. Finally, all these data were analyzed via the one-way ANOVA or Student’s *t*-test where appropriate. *p* < 0.05 was considered to be statistically significant.

## Data Availability

Data is contained within the article or Supplementary Materials.

## Supplementary Materials

The following are available online at https://www.mdpi.com/article/10.3390/ijms22126291/s1.

## Abbreviations

AP1	activator proteins 1
Bcl-2	b-cell lymphoma 2
C/EBP	CCAAT/enhancer-binding protein
CREB	cAMP-response element-binding protein
Cycs	cytochrome c
DMEM	Dulbecco’s Modified Eagle’s Medium
EMSA	electrophoretic mobility shift assay
FBS	fetal bovine serum
FOXO	forkhead box O
HNF1	hepatocyte nuclear factor 1
KLF	Kruppel-like factor
MRE	metal-responsive element
MTF-1	metal-responsive transcription factor 1
PPARα	peroxisome proliferator-activated receptor alpha
RREB1	ras-responsive element-binding protein 1
SP1	specificity protein 1
SREBP	sterol regulatory element-binding proteins
STAT	signal transducer and activator of transcription factor
TFBS	transcription factor binding sites
TSS	transcription start site
Zn	zinc
