# Supplementary material for: Functional Analysis of the Promoter Regions of Two Apoptosis-Related Genes (Bcl-2 and Cycs) and Their Regulation by Zn in Yellow Catfish
Source: Int J Mol Sci. 2021 Jun 11;22(12):6291. doi: 10.3390/ijms22126291 (PMC8230946; doi:10.3390/ijms22126291)
Supplement: Supplementary file 1 [file ijms-22-06291-s001.zip › ijms-1234640-supplementary.pdf]

Table S1. Primers used for 5'-deletion plasmids construction of yellow catfish *Bcl-2* and *Cycs* promoters.

Gene	Primers	Forward primer (5'-3')	Reverse primer (5'-3')
Bcl2	pGl3-534/+96	ctatcgataggtaccgagctcAGTTAAACTACTCAGGGCTATGAGATTC	cagtaccggaatgccaagcttTCCGCAGATTCGCCCATT
	pGl3-906/+96	ctatcgataggtaccgagctcATTAGGTCAGCATGGTAAATGTACTTATT	cagtaccggaatgccaagcttTCCGCAGATTCGCCCATT
	pGl3-1410/+96	ctatcgataggtaccgagctcAACAAGAAAACAATTCCACCACAA	cagtaccggaatgccaagcttTCCGCAGATTCGCCCATT
	pGl3-1990/+96	ctatcgataggtaccgagctcTTAACAGGCACCGTGGACTCA	cagtaccggaatgccaagcttTCCGCAGATTCGCCCATT
Cycs	pGl3-425/+100	ctatcgataggtaccgagctcGAGAGAAGCAGTGGAGAGGGATT	cagtaccggaatgccaagcttCACCGGACGCTACTGCACG
	pGl3-940/+100	ctatcgataggtaccgagctcTCAAGTTTATTTGTATAGCGCTTTTTAC	cagtaccggaatgccaagcttCACCGGACGCTACTGCACG
	pGl3-1410/+100	ctatcgataggtaccgagctcGCACTGAGTTTTCTAAATGGAAGTTAG	cagtaccggaatgccaagcttCACCGGACGCTACTGCACG
	pGl3-1831/+100	ctatcgataggtaccgagctcATATACGGTTCAGTTAGACTGCTGCT	cagtaccggaatgccaagcttCACCGGACGCTACTGCACG

Table S2. Primers used for site-mutation analysis.

Gene	Primers	Forward primer (5'-3')	Reverse primer (5'-3')
Bcl-2	Mut-Bcl-2-MRE	agactgatcagtatgacttgTTTTGCTATATCCTTTGTGCCACT	TggcacatgtgagtcgcTACTGCTCCCTT
	Mut-Bcl-2-HNF-1α	TGcagtctgccagtgtcGCTCTTTAGCGCCTTTTCCA	gacactggcagactgCATTTATAAATAATTTTTAATGACAAACACC
Cycs	Mut-Cycs-MRE	CTgcgcgatctgtatcatTTTCTGCTCACAGAAAAATAAGAAATAA	atgatacagatcgcgcAGTAGCAGATTTTTTTTATTAAACAAACG
	Mut-Cycs-HNF-1α	ActgatggtccctcgcTCAATTCGATTCAATTTAATTCAAGTT	AgcgagggaccatcagTAAATGTCCTTTGTATAGTGCTTTTACC

Table S3. Primers used for electrophoretic mobility-shift assay (EMSA).

Primers		Forward primer (5'-3')	Reverse primer (5'-3')
Bcl2-MRE	Biotin-probe	Biotin-CAGTATTTCAGTGTGCACAATATGT	Biotin-ACATATTGTGCACACTGAAATACTG
	Mutative-competitor	TGACGGCGACTCACATGTGCCGGAC	GTCCGGCACATGTGAGTCGCCGTCA
Bcl2-HNF-1α	Biotin-probe	Biotin-AAATGAGTGAATTAGTAACTGCTCT	Biotin-AGAGCAGTTACTAATTCACTCATTT
	Mutative-competitor	GCCGACAGTCTGCCAGTGTCATCAG	CTGATGACACTGGCAGACTGTCGGC
Cycs-MRE	Biotin-Probe	Biotin-CTACTAATTTGCACAGGGACGTTTC	Biotin-GAAACGTCCCTGTGCAAATTAGTAG
	Mutative-competitor	TGCAGGCGCGATCTGTATCATGCGA	TCGCATGATACAGATCGCGCCTGCA
Cycs-HNF-1α	Biotin-probe	Biotin-ATTTAAGTGAATGATTCATTTCAAT	Biotin-ATTGAAATGAATCATTCACTTAAAT
	Mutative-competitor	GCGCCCTGATGGTCCCTCGCGACGG	CCGTCGCGAGGGACCATCAGGGCGC

Table S4. Primers used for qPCR analysis.

Genes	Forward primer (5'-3')	Reverse primer (5'-3')	Accession No.
Bcl-2	GAGTTCGGTAGCACCCTTTG	AGGGGTGGAATATGGATGCT	XM027173525
Cycs	AGTAGAGAACGGTGGCAAGC	GGGGTTCTCCAAGTACTCCA	KY053836
β-actin	GCACAGTAAAGGCGTTGTGA	ACATCTGCTGGAAGGTGGAC	EU161066
elfa	GTCTGGAGATGCTGCCATTG	AGCCTTCTTCTCAACGCTCT	KU886307
b2m	GCTGATCTGCCATGTGAGTG	TGTCTGACACTGCAGCTGTA	KP938520
hprt	ATGCTTCTGACCTGGAACGT	TTGCGGTTCAGTGCTTTGAT	KP938523
rpl7	GGCAAATGTACAGGAGCGAG	GCCTTGTTGAGCTTGACGAA	KP938522
Ubce	TCAAGAAGAGCCAGTGGAGG	TAGGGGTAGTCGATGGGGAA	KP938524
